# Use of Expedited Regulatory Programs and Clinical Development Times for FDA-Approved Novel Therapeutics

**DOI:** 10.1001/jamanetworkopen.2023.31753

**Published:** 2023-08-31

**Authors:** Alissa K. Wong, Maryam Mooghali, Reshma Ramachandran, Joseph S. Ross, Joshua D. Wallach

**Affiliations:** 1 Section of General Internal Medicine, Department of Internal Medicine, Yale School of Medicine, New Haven, Connecticut; 2 Yale Collaboration for Regulatory Rigor, Integrity and Transparency, Yale School of Medicine, New Haven, Connecticut; 3 Department of Health Policy and Management, Yale School of Public Health, New Haven, Connecticut; 4 Center for Outcomes Research and Evaluation, Yale-New Haven Health System, New Haven, Connecticut; 5 Department of Epidemiology, Rollins School of Public Health, Emory University, Atlanta, Georgia

## Abstract

This cross-sectional study evaluates the duration between application to US Food and Drug Administration (FDA) and approval for new drugs and biologics in the US from 2015 to 2022.

## Introduction

The US Food and Drug Administration (FDA) has 3 regulatory programs intended to expedite both clinical development and FDA’s review of novel therapeutics targeting serious conditions: fast track, accelerated approval, and breakthrough.^1^ Fast track and breakthrough provide opportunities for increased interaction between manufacturers and the FDA as well as rolling review of marketing applications.^1^ Breakthrough and accelerated approval allow for approval based on preliminary clinical evidence, including trials using surrogate markers as primary end points to shorten the duration and size of clinical trials, reducing clinical development times.^1^ A fourth regulatory program, priority review, was designed to expedite the review process from 10 to 6 months. Previous studies have evaluated the implications of the FDA’s expedited regulatory programs for the combined clinical development and review times for novel therapeutics, but their findings have been inconsistent and have not focused on clinical development times, the target of these 3 programs.^2,3^ Given their growing use,^4^ we assessed whether expedited regulatory programs were associated with faster clinical development times for FDA-approved novel therapeutics.

## Methods

In accordance with the Common Rule, this cross-sectional study was exempt from ethics review and informed consent because it used public, nonidentifiable data and was not human participant research. We followed the STROBE reporting guideline.

We used the FDA Novel Drug Summary to identify all new drugs and biologics that were approved between January 2015 and December 2022 and their corresponding expedited regulatory programs. Drugs@FDA was used to identify Investigational New Drug (IND) application dates, corresponding with the start of human testing, and New Drug Application (NDA) or Biologics License Application (BLA) submission and approval dates. For therapeutics without IND application dates on Drugs@FDA, we searched patent extension notices using the *Federal Register*, ClinicalTrials.gov, or Google for these dates or phase-1 study start dates. For therapeutics with multiple formulations, we selected the earliest IND application dates. Clinical development times and combined clinical development and review times were calculated as the time from IND application to NDA or BLA submission and approval, respectively, and were compared across expedited regulatory program used (primary analysis: ≥1 vs none; secondary analyses: fast track, accelerated approval, or breakthrough vs none) along with therapeutic type and area.

Analysis was performed using Mann-Whitney tests in RStudio (Posit). Two-tailed *P* < .05 were considered statistically significant.

## Results

The FDA approved 367 therapeutics between 2015 and 2022, of which 7 had multiple formulations, 6 were developed outside of the US, and 14 had no publicly available IND dates. Of the remaining 340 approvals, 196 (57%) used at least 1 expedited regulatory program: 118 (35%) fast track, 64 (19%) accelerated approval, and 109 (32%) breakthrough.

Median (IQR) clinical development times for approvals using 1 or more vs no expedited regulatory programs were 6.0 (4.0-8.2) vs 7.2 (5.3-9.5; *P* = .001) years, respectively (Table 1). Median (IQR) combined clinical development and review times were 7.0 (4.8-9.0) and 8.3 (6.5-11.4; *P* < .001) years, respectively, for 1 or more vs no expedited regulatory programs (Table 2). Differences were consistently statistically significant only for cancer and hematology and other category therapeutics. Compared with approvals using no expedited program (7.2 [5.3-9.5] years), approvals using accelerated approval (4.9 [4.0-7.4] years; *P* < .001) and breakthrough (5.2 [3.8-7.6] years; *P* < .001) had significantly shorter median (IQR) clinical development times, but not fast track (6.6 [4.2-9.3] years; *P* = .10).

**Table 1.** Duration Between Investigational New Drug Application and New Drug Application (NDA) or Biologics License Application (BLA) Submission Date for Novel Therapeutics Approved by the Food and Drug Administration From 2015 to 2022

	Total No. (%)	Approvals using no expedited programs		Approvals using ≥1 expedited programs		*P* value
		No. (%)	Median (IQR), y	No. (%)	Median (IQR), y	
Overall	340 (100)	144 (42.4)	7.2 (5.3-9.5)	196 (67.6)	6.0 (4.0-8.2)	.001
Therapeutic type						
BLA	96 (28.2)	33 (34.4)	7.5 (5.7-10.1)	63 (65.6)	6.5 (4.0-8.3)	.02
NDA	244 (71.8)	111 (45.5)	7.0 (5.2-9.0)	133 (54.5)	5.9 (4.1-8.2)	.008
Therapeutic area						
Cancer and hematology	120 (35.3)	28 (23.3)	6.9 (5.7-11.4)	92 (76.7)	5.7 (4.0-7.9)	.007
Infectious disease	44 (12.9)	9 (20.5)	7.7 (2.3-9.1)	35 (79.5)	8.0 (3.6-10.4)	.49
CVD, diabetes, and hyperlipidemia	22 (6.5)	14 (63.6)	5.4 (4.4-8.1)	8 (26.3)	7.5 (6.7-10.9)	.19
Autoimmune, musculoskeletal, and dermatology	41 (12.1)	25 (61.0)	6.5 (5.5-7.7)	16 (39.0)	5.9 (4.6-7.2)	.14
Neurology and psychiatry	46 (13.5)	30 (65.2)	7.5 (6.0-8.7)	16 (34.8)	5.2 (4.4-7.5)	.07
Other^a^	67 (19.7)	38 (56.7)	7.3 (5.0-10.7)	29 (43.3)	6.0 (4.0-7.5)	.03

Abbreviation: CVD, cardiovascular disease.

^a^ Other included endocrinology (excluding diabetes) and metabolic disorders; gastroenterology; gynecology; nephrology; opthalmology; pulmonology; and therapeutics for the treatment of amyloidosis, prevention of nausea and vomiting (after surgery or delayed phase chemotherapy–induced), induction and maintenance of procedural sedation, reversing effects of neuromuscular blocking drugs during surgery, treatment of submental fat, and management of acute pain.

**Table 2.** Duration Between Investigational New Drug Application and New Drug Application (NDA) or Biologics License Application (BLA) Approval Date for Novel Therapeutics Approved by the US Food and Drug Administration From 2015 to 2022

	Total No. (%)	Approvals using no expedited programs		Approvals using ≥1 expedited programs		*P* value
		No. (%)	Median (IQR), y	No. (%)	Median (IQR), y	
Overall	340 (100)	144 (42.4)	8.3 (6.5-11.4)	196 (57.6)	7.0 (4.8-9.0)	<.001
Therapeutic type						
BLA	96 (28.2)	33 (34.4)	8.4 (7.1-11.4)	63 (65.6)	7.2 (4.8-9.1)	.007
NDA	244 (71.8)	111 (45.5)	8.1 (6.4-11.1)	133 (54.5)	6.9 (4.7-9.0)	<.001
Therapeutic area						
Cancer and hematology	120 (35.5)	28 (23.3)	7.7 (6.5-12.0)	92 (76.7)	6.3 (4.7-8.6)	.004
Infectious disease	44 (12.9)	9 (20.5)	9.5 (2.8-11.9)	35 (79.5)	8.7 (4.5-11.2)	.75
CVD, diabetes, and hyperlipidemia	22 (6.5)	14 (63.6)	7.0 (6.0-9.0)	8 (36.4)	8.2 (7.6-12.-0)	.33
Autoimmune, musculoskeletal, and dermatology	41 (12.1)	25 (61.0)	7.8 (6.9-8.7)	16 (39.0)	6.7 (5.0-8.1)	.07
Neurology and psychiatry	46 (13.5)	30 (65.2)	8.7 (6.9-11.2)	16 (34.8)	6.0 (4.6-8.3)	.02
Other^a^	67 (19.7)	38 (56.7)	8.5 (6.8-11.9)	29 (43.3)	7.2 (4.7-8.6)	.007

Abbreviation: CVD, cardiovascular disease.

^a^ Other included endocrinology (excluding diabetes) and metabolic disorders; gastroenterology; gynecology; nephrology; opthalmology; pulmonology; and therapeutics for the treatment of amyloidosis, prevention of nausea and vomiting (after surgery or delayed phase chemotherapy–induced), induction and maintenance of procedural sedation, reversing effects of neuromuscular blocking drugs during surgery, treatment of submental fat, and management of acute pain.

## Discussion

We found that FDA approvals using expedited regulatory programs had shorter clinical development times and combined clinical development and review times by approximately 1 to 2 years, demonstrating the association of these programs with faster therapeutic clinical testing and approval. Study limitations included the reliance on publicly available IND submission dates, which were not uniformly reported. Given that expedited regulatory programs often facilitate faster approval through postmarketing studies required to address residual clinical uncertainties, ongoing efforts are needed to ensure that these studies are completed and reported in a timely manner.^5,6^

## Notes

Supplement.
Data Sharing Statement
