# Supplementary material for: Use of Expedited Regulatory Programs and Clinical Development Times for FDA-Approved Novel Therapeutics
Source: JAMA Netw Open. 2023 Aug 31;6(8):e2331753. doi: 10.1001/jamanetworkopen.2023.31753 (PMC10472182; doi:10.1001/jamanetworkopen.2023.31753)
Supplement: Supplement. — Data Sharing Statement [file jamanetwopen-e2331753-s001.pdf]

Data Sharing Statement

Wong. Use of Expedited Regulatory Programs and Clinical Development Times for FDA-Approved Novel Therapeutics. *JAMA Netw Open*. Published August 31, 2023. doi:10.1001/jamanetworkopen.2023.31753

Data
Data available: Yes
Data types: Data (not involving human participants)
How to access data: Data available upon request upon publication: joshua.wallach@emory.edu
When available: With publication

Supporting Documents
Document types: None

Additional Information
Who can access the data: Anyone requesting the data
Types of analyses: For any purpose
Mechanisms of data availability: Without investigator support
Any additional restrictions: None
